# A survey exploring factors affecting employment of physician associates in Ireland

**DOI:** 10.1007/s11845-022-03255-9

**Published:** 2023-01-05

**Authors:** Pauline Joyce, Lisa Alexander

**Affiliations:** RCSI: Royal College of Surgeons in Ireland, Dublin, Ireland

**Keywords:** Barriers, Employment, Influences, Physician associate

## Abstract

**Background:**

In the Republic of Ireland, the employment of physician associates (PAs) is growing. Following a pilot project in a hospital setting, PAs are now employed across primary and secondary care in public and private sectors. Most of the Irish PA graduates are working in hospital settings.

**Aims:**

The aim of the study was to explore factors which supported or inhibited the employment of PAs in Irish hospital settings and the perceived supports or challenges for potential employers in recruiting PAs.

**Methods:**

An online survey gathered data via human resources departments of public and private hospitals, with a 25% response rate.

**Results:**

Similar to previous studies, the barriers included the lack of recognition and regulation of the role and the small number of PAs to fill available posts. Enablers, which influenced the employment of PAs, included improving workflow, continuity of care and helping to address junior doctors’ working hours.

**Conclusions:**

Our data suggests that there is a keen interest and willingness to employ PAs and there is great potential to expand the role in Irish healthcare. However, there are some key issues around funding and recognition to be addressed at government level for this profession to highlight its worth.

## Background

The first physician associates (PAs) were employed in Ireland in 2018 following a pilot project with the Department of Health between 2015 and 2017 [1]. From the 52 Irish-trained PA graduates working in the Irish healthcare system, 36 were working in public hospitals, 4 in primary GP settings and 12 in private settings, at the time of this study. It was therefore opportune to explore what factors supported or challenged their employment. For hospitals who have not yet recruited PAs, it was important to understand the potential perceived challenges or supports when considering employing a PA. Ultimately, the rationale for undertaking this research was to explore the perceived demand for the PA role, and, as the only university in Ireland educating this group of professionals, to plan for an increase in student number if required. As with some other countries, the PA profession is not yet regulated in Ireland, something which has been reported as a barrier for potential employers because of indemnity issues [2]. Halter et al. [3] sampled medical directors of all acute and mental health trusts in England, to explore employment factors there. Some had already employed PAs whilst others might have considered employing PAs but highlighted some barriers to this process. The most common motivators for employing PAs, according to their study, were those related to filling a gap in medical staffing, improving workflow, reducing staff costs and supporting medical trainees. Substituting for residents in the face of medical staffing has been highlighted in the USA as a common reason for hiring PAs [4]. In addition, Halter et al. [3] found that inhibiting factors to employment of PAs included the lack of regulation, as yet, for PAs in the UK, meaning that the PAs cannot yet prescribe or order ionising radiation. They also found that there were not enough PA graduates to recruit.

## Methods

This descriptive study explored the factors which supported or inhibited the employment of PAs and the perceived supports or challenges for potential employers in recruiting PAs. The survey link was sent to available human resource (HR) contacts in all public (*n* = 86) and private (*n* = 18) hospitals in Ireland. However, 10 emails from the public hospitals and 2 from the private hospitals were undelivered due to out-of-date contact addresses available. It was deemed important to distinguish the public and private interest in employing PAs because some private sectors were already providing scholarships for PA students to undertake the programme with a contract to work in their organisation on qualification. The fees have proven a challenge for some students as the intensity of the programme does not allow them to take on part-time work. GP settings were not included in the survey due to the low number of PAs (four, at time of writing) employed in this setting, and hence the possibility of identification. Once participants agreed to participate in the survey, an anonymous survey link generated by the RCSI Quality Enhancement Office was emailed to distribute to HR staff at their hospital settings, for example business managers, who are involved or could be involved at a future date in employment of PAs. The study received ethical approval and included a consent form. Data was collected and analysed via SurveyMonkey and open comments were analysed via content analysis for themes.

A validated survey [3], amended to suit the Irish setting, was used. The survey comprised 10 questions, some having closed, single or multiple response options with an opportunity for open comments and one question having an open response option only. The rationale for using a survey was to compare findings from Ireland with the UK and to target a larger sample, than could be done at interview. However, one-to-one conversations have also been ongoing with potential employers as part of the marketing of the programme.

## Results

**Fig. 1:**
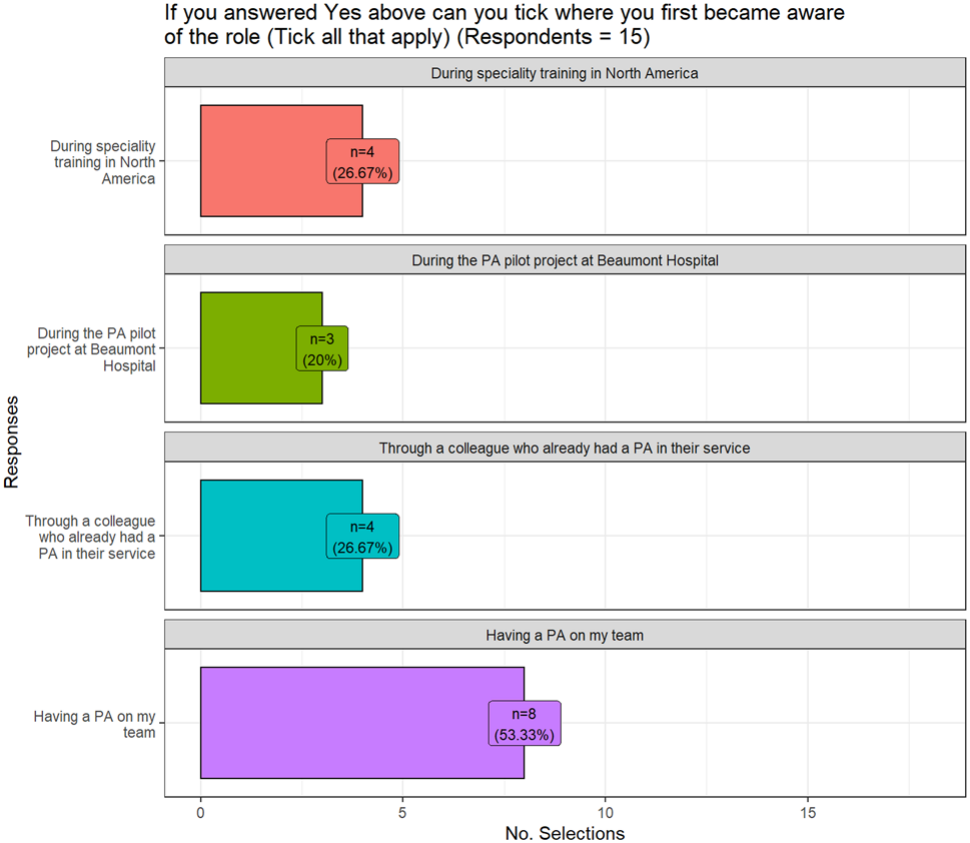
Reasons for familiarity with PA role

Response rate was calculated at 25%, *n* = 17 from the public hospitals and *n* = 6 from private hospitals. Of the sample surveyed, 92% of respondents were already familiar with the PA role, with the most common reason for the familiarity being the employment of a PA on the team. The next most common reason for being familiar with the role was their training experience in North America or from a colleague who had a PA on their team (Fig. 1). Additional responses in the free text comments section included the awareness of the role through RCSI, the programme team, the PA pilot project, and having students rotate through the service (Table 1).

**Table 1:** Additional comments on employing a PA

Themes	Example of comment
1. Funding and salary	Identification of a funding stream for the PA is essential to encourage engagement and employment at hospital level
	There needs to be a salary scale and progression
2. Regulation and recognition	Without IMC* recognition for the PA role in Ireland, it is very difficult to employ this grade in the public setting as part of a medical team
	Lack of role recognition is the major barrier. Getting this recognised would make PAs very attractive for reasons of continuity of care and effective clinical service provision
3. Available PA graduates	We see the main current limitation as the small number of PAs graduating per year, understanding that fees for candidates are a limiting factor
	Our Consultant Lead would like a PA for each discipline in all fields in our hospital. They are highly sought after and excellent feedback at Management Team Meetings

*IMC Irish Medical Council—the regulator for doctors in Ireland

**Fig. 2:**
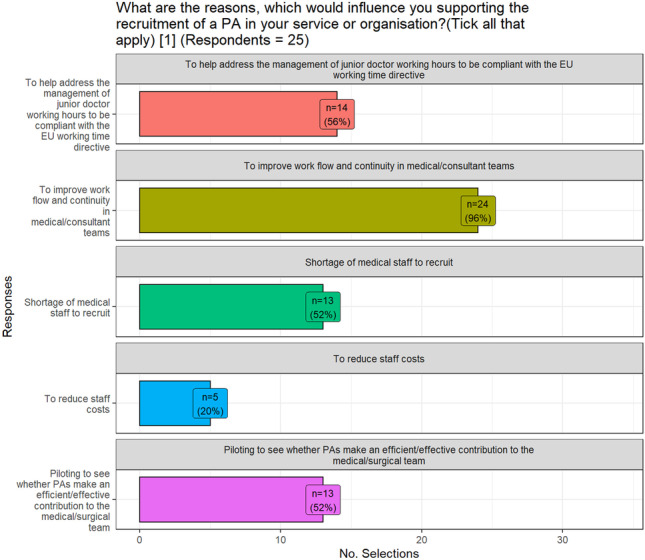
Influences supporting PA employment

Whilst 36% of the sample did not have any PAs employed in their organisation, 60% of the respondents had between one and ten PAs employed. The influences supporting the employment of PAs varied, with 96% selecting the option ‘To improve work flow and continuity in medical/consultant teams’ (Fig. 2). Interestingly, the reduction in staff costs had the least influence in this decision. Additional reasons supplied included the requirement to have a permanent member on the medical team, who was not on rotation, to address many workforce issues in surgical practice, and to permit all staff to work to the highest level of their licencing.

**Fig. 3:**
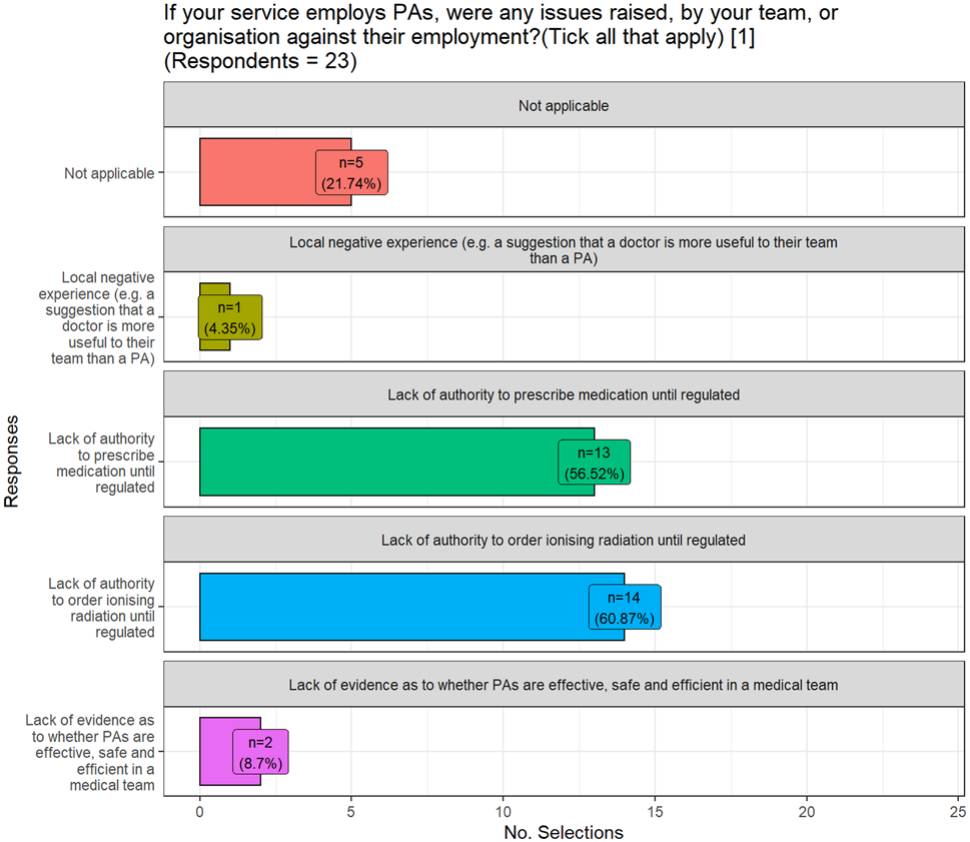
Barriers to recruiting PAs

When exploring the barriers to recruiting PAs, the lack of regulation for PAs currently in Ireland had a clear influence. Due to this lack of regulation, PAs are not permitted to order ionising radiation or prescribe medications. Figure 3 shows that this situation was a deciding factor for the team or organisation. Additional comments for this question included financial constraints, lack of experience with the PA role, and what they are permitted to do, and concerns from nursing and junior doctors.

Further issues which were raised when recruiting PAs included the lack of PAs available (27%) with some (13%) identifying opposition from other groups which may include junior doctors and nurses. Where there were previous posts filled by a PA who subsequently left the post (*n* = 4), the reason for three of these posts remaining unfilled was the unsuccessful employment of a PA, relating back to the low numbers of graduates to date. One respondent suggested that the consultant decided on replacing the post with a doctor.

The final question in the survey allowed respondents to provide any additional comments about employment of PAs in their organisations. Clearly, the barriers of lack of salary benchmarking, regulation and the small number of PAs available are to the forefront here (Table 1).

## Discussion

According to Raffoul [5], the quality of any healthcare system depends on the calibre, enthusiasm and diversity of the workforce. The PA workforce is expanding globally, with two main factors driving its growth, i.e. increasing access to care and providing continuity of care [6, 7]. Strategies to increase access to care have been influenced by a global shortage of doctors. According to Cawley and Hooker [8], the introduction of PAs has succeeded in some countries more than others with these successes attributed to social and professional acceptance. The majority of the sample in this study were familiar with the PA role due to the employment of a PA on the team. Familiarity can be a key influence in attitude to the PA role and the willingness of healthcare professionals to work with PAs [9]. Despite the PA role being in place for over 50 years in the USA and the increasing popularity of the profession globally, Volpe et al. [10] found that college students were unfamiliar with the PA role. However, when medical students were familiar with the role, they indicated a willingness to work with the PA in referring patients to them, if this would reduce wait time for patients [11]. A common reason for familiarity with the PA role included the respondent’s experience of the PA during speciality training in North America [2]. With training for new initiatives such as robotic surgery continuing to be popular in the USA [12], more Irish doctors could become familiar with the PA role.

Improving workflow and continuity in medical teams was the most popular influencing factor chosen for recruiting a PA, chosen in this study. In previous studies [1–3, 13–16], the quality and the continuity of care, particularly at the time of trainee doctors’ change-over, improved, according to staff in the hospital, with the introduction of the PA role, allowing the PA to be a reliable point of contact for other departments, facilitating communication across the hospital. PAs generally do not rotate and can be a more stable factor in the continually changing medical workforce, and are thought to be more familiar with the routines of other individual professionals [13]. In addition, PAs, in this study, were judged to improve work flow, concurring with previous studies in their ability to increase the capacity of the team [14, 17]. Moreover, studies have found that patients are satisfied with the care they receive from PAs [18–21].

Where there is lack of experience with the PA role and concerns from other healthcare professionals, Timmermans [13] suggests that mutual trust emerged as a perceived facilitator for sustaining the employment of PAs. Although their study was carried out 15 years after the introduction of the PA role in the Netherlands, their findings suggest that PAs experienced resistance in varying strengths, for example resistance from physicians who do not want to consult a PA, and from residents who think that the employment of PAs interferes with their job and education possibilities [13]. Another Dutch study [22] found lack of team support when introducing PAs in the primary care settings and recommend long-term political planning and support when introducing such roles. Whilst the reduction in staff costs had the least influence in a decision to employ a PA, in some countries the PA growth may be linked with top-down, long-term investment, to ensure a sustainable programme for PAs [8]. Given the large average salary difference between PAs and doctors, a shift towards PAs could have the direct effect of lowering healthcare costs [23].

Whilst shortages in the healthcare workforce are anticipated worldwide, Bohmer and Imison [24] believe that clarity about roles and responsibilities is critical to the successful implementation of workforce redesign. Together with role clarity comes task shifting, now becoming a common strategy for healthcare reform in many countries. In the USA, organisations are actively including PAs in their workforce planning strategies [25]. There is support for leveraging the talents of both PAs and Nurse Practitioners (NPs) with the expectation of a growth in PAs alone by 30% in the USA by 2030 [26].

The lack of regulation of the PA role is viewed as a barrier in the UK and in other countries to the employment of PAs [3, 27–29]. Yet, it has been suggested that PAs have demonstrated that they are clinically safe, competent and surrounded by a vigorous governance and quality assurance, despite a lack of regulation [30]. On the other hand, PAs, as advanced practice providers, may be hampered in practicing to their full potential due to the absence of regulation. The Ministry of Health, Welfare and Sports, in the Netherlands, has adopted a national task shifting policy to grant full practice authority (FPA), specifically for the performance of cardioversion/defibrillation, catheterisation, endoscopy, injections, prescribing, puncture and small surgical procedures to their NPs and PAs [31]. Their study findings suggest that the granting of FPA to their advanced practice providers increased the effectiveness of care delivery. PAs can provide a flexible addition to the primary and secondary care workforce without drawing on other professions. In fact, a key element of Sláintecare [32] is shifting the focus of care delivery from a hospital-centric model to one with greater access to care delivery in the community. A recent ESRI report [33] raises important considerations for policymakers in terms of acute workforce investment, workforce planning and training both nationally and regionally over the coming years.

Limitations of this study include its small sample size and response rate. Despite two email reminders about the survey, the number of responses did not improve greatly. In addition, the survey only contained ten questions. A mixed methods study could have probed some of the responses to gain a clearer picture of any suggestions to encourage employment of this new role and could identify specific challenges that could be addressed. Launching a study such as this one at a workforce planning meeting or conference might encourage more of the sample to respond.

## Conclusion

Although a snapshot in time, in the early phase of introducing PAs in the Republic of Ireland, the data concurs with the barriers and influences in recruiting PAs, found in previous studies. Whilst 92% of the samples were familiar with the PA role, this was because of either having a PA on their medical team or having carried out some of their medical training in North America, where the PA role is embedded into the medical team. Improving the workflow and continuity of patient care were top priorities for employing a PA, with financial cost being the least influencing factor. The lack of regulation for the PA role was clearly a barrier for some employers, as the resultant consequences include the lack of prescribing rights. Lesser barriers include the lack of experience of the PA role and uncertainty about their scope of practice. These concerns were perceived to be shared by some nursing colleagues and junior doctors. The supply of PA graduates not meeting demand was also raised as a barrier, with some posts remaining unfilled. In addition, the benchmarking of salary for this new profession in Ireland was included in the additional comments section, as needing to be progressed. Further research could be carried out using one-to-one interviews to probe some of the responses from this small study and to explore if there are different influences and barriers across the public and private sectors and across urban and rural areas. When employment of the PA role is increased in primary care, data from these employers would also enrich the study.

## Data Availability

Data from this survey is available.

## References

[1] Joyce P, Hooker RS, Woodmansee D, Hill AD (2019). Introducing the physician associate role in Ireland: evaluation of a hospital based pilot project. J Hosp Adm.

[2] Hix LR, Fernandes SM, Joyce P (2020). Experience of the Irish physician associate role: PA and supervising consultant perspectives. Int J Healthc.

[3] Halter M, Wheeler C, Drennan VM, de Lusignan S, Grant R, Gabe J (2017). Physician associates in England’s hospitals: a survey of medical directors exploring current usage and factors affecting employment. Clin Med.

[4] Morgan P, Everett CM, Humeniuk KM, Valentin VL (2016). Physician assistant specialty choice: distribution, salaries, and comparison with physicians. J Am Acad PAs.

[5] Raffoul M, Bartlett-Esquilant G, Phillips RLJ (2019). Recruiting and training a health professions workforce to meet the needs of tomorrow’s health care system. Acad Med.

[6] Harkins T, Thomas J, Fontenot B, Day J, Faraone M, Borden A et al (2021) Utilization and workforce integration of physician assistants. 19

[7] Taylor M, Taylor DW, Burrows K, Cunnington J, Liou M (2013) Qualitative study of employment of physician assistants by physicians. Can Fam Physician 59:e507 PMC3828112 24235209

[8] Cawley J, Hooker R (2018). Determinants of the physician assistant/associate concept in global health systems. Int J Healthc.

[9] Kreeftenberg HG, Aarts JT, Kerkhoven C, Rosmalen van J, Bindels AJGH, van der Voort PHJ (2022) A survey on implementation of physician assistants in ICUs in the Netherlands. 30(4)

[10] Volpe M, Bulmer S, Kelsey C (2015) Knowledge and perceptions of college students regarding the physician assistant profession. Cureus [Internet]. [cited 2022 Jul 20] 7(10). Available from: https://www.cureus.com/articles/3486-knowledge-and-perceptions-of-college-students-regarding-the-physician-assistant-profession 10.7759/cureus.368 PMC4659584 26623223

[11] Joyce P, Syed S, Arnett R, Sreenan S, Hooker RS (2020). Willingness of medical students to refer patients to a physician associate or a doctor based on clinical scenarios when time is a trade-off. Int J Healthc.

[12] O’Connell LV, Hayes C, Ismail M, O’Ríordáin DS, Hafeez A (2022). Attitudes and access of Irish general surgery trainees to robotic surgical training. Surg Open Sci.

[13] Timmermans MJC, van Vught AJAH, Maassen ITHM, Draaijer L, Hoofwijk AGM, Spanier M (2016). Determinants of the sustained employment of physician assistants in hospitals: a qualitative study. BMJ Open.

[14] Imison C, Castle-Clarke S, Watson R (2016) Reshaping the workforce to deliver the care patients need [Internet]. Nuffield Trust. [cited 2022 Jul 13] p. 92. (Evidence for Better Healthcare). Report No.: ISBN: 978–1–910953–11–2. Available from: www.nuffieldtrust.org.uk

[15] Drennan VM, Halter M, Wheeler C, Nice L, Brearley S, Ennis J (2019). What is the contribution of physician associates in hospital care in England? A mixed methods, multiple case study. BMJ Open.

[16] van Vught AJAH, van den Brink GTWJ, Wobbes T (2014). Implementation of the physician assistant in Dutch health care organizations: primary motives and outcomes. Health Care Manag.

[17] Halter M, Wheeler C, Pelone F, Gage H, de Lusignan S, Parle J (2018). Contribution of physician assistants/associates to secondary care: a systematic review. BMJ Open.

[18] Joyce P (2019) Patient satisfaction with care as managed by the physician associate or the doctor as part of a pilot project in Ireland. J Health Med Sci [Internet]. [cited 2022 Feb 3] 2(2). Available from: https://www.asianinstituteofresearch.org/JHMSarchives/Patient-Satisfaction-with-Care-as-Managed-by-the-Physician-Associate-or-the-Doctor-as-Part-of-a-Pilot-Project-in-Ireland

[19] Kurtzman ET, Barnow BS (2017). A comparison of nurse practitioners, physician assistants, and primary care physicians’ patterns of practice and quality of care in health centers. Med Care.

[20] Meijer K, Kuilman L (2017). Patient satisfaction with PAs in the Netherlands. JAAPA.

[21] Hooker RS, Moloney-Johns AJ, McFarland MM (2019). Patient satisfaction with physician assistant/associate care: an international scoping review. Hum Resour Health.

[22] van der Biezen M, Derckx E, Wensing M, Laurant M (2017). Factors influencing decision of general practitioners and managers to train and employ a nurse practitioner or physician assistant in primary care: a qualitative study. BMC Fam Pract.

[23] Walia B, Banga H, Larsen DA (2022). Increased reliance on physician assistants: an access-quality tradeoff?. J Mark Access Health Policy.

[24] Bohmer RMJ, Imison C (2013). Lessons from England’s health care workforce redesign: no quick fixes. Health Aff (Millwood).

[25] Zaletel CL, Madura B, Metzel JM, Lancaster RJ (2022). Optimizing the productivity and placement of NPs and PAs in outpatient primary care sites. JAAPA.

[26] US Department of Labor, Bureau of Labor Statistics (2022) Physician assistants : occupational outlook handbook: : U.S. Bureau of Labor Statistics [Internet]. [cited 2022 Aug 2]. Available from: https://www.bls.gov/ooh/healthcare/physician-assistants.htm

[27] Hix LR, Fernandes SM (2020). An initial exploration of the physician assistant role in Germany. J Physician Assist Educ.

[28] Malone R (1971) The role of the physician associate: an overview. Ir J Med Sci 191(3):1277–83 10.1007/s11845-021-02661-9 34351601

[29] Williams LE, Ritsema TS (2014). Satisfaction of doctors with the role of physician associates. Clin Med.

[30] Aiello M, Roberts KA (2017). Development of the United Kingdom physician associate profession. JAAPA.

[31] Bruijn-Geraets DPD, van Eijk-Hustings YJL, Bessems-Beks MCM, Essers BAB, Dirksen CD, Vrijhoef HJM (2018). National mixed methods evaluation of the effects of removing legal barriers to full practice authority of Dutch nurse practitioners and physician assistants. BMJ Open.

[32] gov.ie - Sláintecare [Internet]. [cited 2022 Aug 8]. Available from: https://www.gov.ie/en/campaigns/slaintecare-implementation-strategy/

[33] Keegan C, Brick A, Rodriguez AG, Hill L (2022) Projections of workforce requirements for public acute hospitals in Ireland, 2019–2035:a regional analysis based on the Hippocrates model [Internet]. ESRI [cited 2022 Aug 3]. Available from: https://www.esri.ie/publications/projections-of-workforce-requirements-for-public-acute-hospitals-in-ireland-2019-2035a

